# Laparoscopic Sleeve Gastrectomy versus Lifestyle Modification in Class I Obesity in Pakistani Population: A Prospective Cohort Study

**DOI:** 10.7759/cureus.5031

**Published:** 2019-06-28

**Authors:** Amina Amin, Ghulam Siddiq, Muhammad Ijlal Haider, Usama Khalid Choudry, Izza Nazir

**Affiliations:** 1 General Surgery, Shifa International Hospital, Islamabad, PAK

**Keywords:** laparoscopic sleeve gastrectomy, weight loss program, diabetes mellitus, hypertension

## Abstract

Introduction

The American Society of Metabolic and Bariatric Surgery has stated that bariatric surgery is indicated in Class I obesity patients with one or more comorbidities. However, other weight loss options, such as diet plus exercise, are available to patients with a body mass index (BMI) ranging from 30 to 35 kg/m^2^. This study aimed to prospectively compare the results of Class I obesity patients undergoing laparoscopic sleeve gastrectomy (LSG) or using a weight control program (WCP).

Methods

A prospective analysis was conducted of patients with Class I obesity and comorbid diabetes and hypertension, with follow-ups at 6, 12, and 18 months. Subjects were divided into two groups: the LSG group of patients who had undergone LSG, and the WCP group who adhered to a WCP. The percentage of excess BMI loss (%EBMIL) and comorbidity remission (diabetes mellitus and hypertension) were tracked with measurements of hemoglobin A1C (HBA1C) levels and systolic blood pressure. Self-esteem was also tracked using the Rosenberg Self-Esteem Scale (SES) at 0 and 18 months. The overall patient satisfaction score was calculated using a visual analogue scale.

Results

Of the 150 patients enrolled in the study, 106 were included in the LSG group, and 103 were included in the WCP group. The reduction in HBA1C was more pronounced in the LSG group, and the differences between the two were statistically significant after 6, 12, and 18 months (LSG 5.6 ± 0.47 vs. WCP 6.5 ± 0.64, CI 1.04-0.73, P < 0.05). At 12 and 18 months, there were statistically significant reductions in systolic blood pressure after LSG (LSG 134.2 ± 7.16 vs. WCP 145.63 ± 5.94, CI 13.2-9.6, P < 0.05). Self-esteem levels measured by the Rosenberg SES increased for all participants, while patient satisfaction score was higher in the LSG group than that in the WCP group (P < 0.05). The %EBMIL at 6 months in the LSG group was 35.48%, compared to the WCP group at only 7.23%. At 12 months, the %EBMIL had increased twofold in the LSG group, at 68.19%, compared to 14.53% in the WCP group. At the final 18-month follow-up, the %EBMIL in the LSG group was 99.60% but was only 25.70% in the WCP group (P < 0.05).

Conclusion

Our study elucidates a clear superiority of LSG over any structured WCP with regard to weight reduction, improvement in glycemic control, and reduction in blood pressure in Class I obesity patients. Additionally, patients having LSG reported markedly improved self-esteem and satisfaction when compared with those who undertook a WCP.

## Introduction

Pakistan is ranked as the ninth most obese nation in the world, leading to a public health concern. Obesity is further characterized into three classes based on body mass index (BMI), where Class I obesity encompasses those with a BMI in the range of 30 through 34.9 kg/m^2^. According to the World Health Organization (WHO), in 2016, more than 1.9 billion adults aged 18 years and older were overweight. Of these, over 650 million adults were obese. In the United States, the number of obese adults increased two-fold between 1980 and 2010 (from 16% to 36.1%) [1]. Obesity predisposes individuals to the risk of metabolic conditions such as diabetes mellitus, hypertension, and cardiovascular disease at a younger age [2]. The goal in treating obesity is to improve quality of life and to increase life expectancy by countering the life-shortening effects of obesity and its associated comorbidities [3]. There are various methods of combatting obesity, ranging from lifestyle modifications, such as dietary modifications and physical activity, to various metabolic surgeries [4]. Bariatric surgery has evolved in recent decades, and today, new procedures such as sleeve gastrectomy and Roux-en-Y gastric bypass are more commonly utilized than older methods such as duodenal switch, biliopancreatic diversion and gastric band. However, the effects of bariatric surgical procedures on weight loss, hormonal function, and metabolic parameters are still being researched [5]. In this study, we compared the outcomes at one year of patients who underwent laparoscopic sleeve gastrectomy (LSG) and patients who chose lifestyle modification as a modality for weight loss and issues related to obesity.

## Materials and methods

A prospective cohort study was completed with patients who had a BMI between 30 and 35 kg/m^2^. Written and informed consent was received from all participants. The subjects were divided into two groups: the LSG group, which included Class I obesity patients undergoing LSG, and the weight control program (WCP) group, which included Class I obesity patients who undertook a WCP for a minimum duration of 18 months without any gaps. The sample size was calculated as 150 subjects per group. All subjects had follow-ups at 6, 12, and 18 months, and patients who did not complete the follow-ups were excluded from the study. The WCP included dietary modification and daily exercise, recommended and overseen by a nutritionist, where the initial recommendations were a diet of 1300 to 1600 kcal/day and exercise for at least 45 min 5 days per week. Daily caloric intake and exercise regimens were accordingly graduated with weight loss results at follow-ups. The main goal of the WCP was to instill healthier behaviors in individuals with Class I obesity.

The primary comparative measure of the study’s outcome was the percentage of excess BMI loss (%EBMIL), and the secondary comparisons of the outcome included measurements of hemoglobinA1C (HBA1c) levels, systolic blood pressure, Rosenberg Self-Esteem Scale (SES) scores, and patient satisfaction ratings. %EBMIL was calculated as (initial BMI−current BMI)/(initial BMI−25)*100. Ideal weight was determined to be a BMI of 25 kg/m2. Patients were advised to stop taking antihypertensive medications (if any) 7 days before follow-ups, and HBA1c levels were collected one day before follow-ups. Self-esteem was measured using the Rosenberg SES, which is a standard scale for quantifying self-esteem, and patient satisfaction score was calculated using a visual analogue score. Data were expressed as mean ± standard deviation (SD), and effect modifiers were controlled through stratification. An independent sample T-test was applied for comparison between the groups, and the signiﬁcance level was determined to be < 0.05. Analysis was completed using SPSS software, Version 22 (IBM Inc., Armonk, NY, USA).

## Results

**Figure 1:**
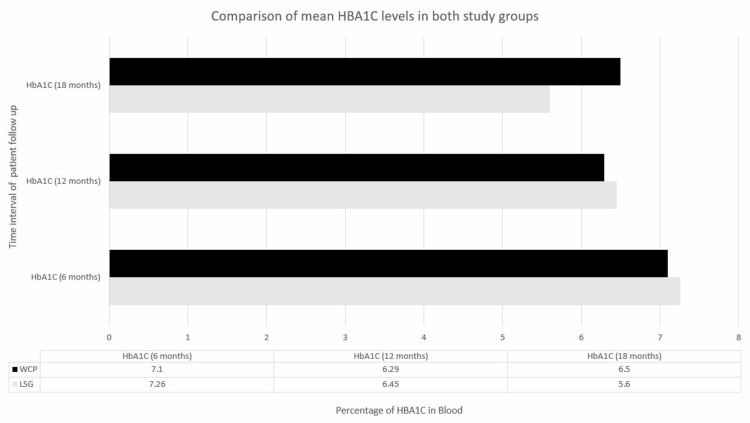
Comparison of mean HBA1C in both study groups HBA1C : Hemoglobin A1C; WCP : Weight Control Program; LSG : Laparoscopic Sleeve Gastrectomy

Out of 300 patients, 91 were excluded due to loss at follow-up. Of the remaining 209 patients, 106 were included in the LSG group, and 103 were included in the WCP group. The study included 69.9% (n = 146) females, while 36% (n = 63) were males. The mean preoperative levels were as follows: HBA1c was 7.96 ± 0.82% in the LSG group and 7.57 ± 0.65% in the WCP group; systolic blood pressure was 165 ± 10.4 mmHg in the LSG group and 162 ± 11.32 mmHg in the WCP group; BMI was 33.40 ± 1.03 kg/m2 in the LSG group and 32.57 ± 1.15 kg/m^2^ in the WCP group; and the Rosenberg SES was 15.53 ± 3.39 in the LSG group and 14.77 ± 2.64 in the WCP group. At 6-month follow-up, mean BMI was 30.47 ± 1.01 kg/m^2^ in the LSG group, while in the WCP group, it was 32.04 ± 1.01 kg/m^2^ (CI 1.85-1.29) (P > 0.05). A statistically significant difference was also found at 12 months and 18 months with LSG 27.67 ± 1.04 versus WCP 31.47 ± 0.92, CI 4.11-3.47, P < 0.05 and LSG 25.03 ± 1.47 versus WCP 30.62 ± 1.02, CI 5.94-5.24, P < 0.05, respectively. There was a significant reduction in mean HBA1c levels in both groups; however, the reduction was more pronounced in the LSG group, and the difference between the two was statistically significant after 6, 12, and 18 months, at LSG 7.26 ± 0.54 versus WCP 7.10 ± 0.59, CI 0.04-0.31, P = 0.04; LSG 6.45 ± 0.40 versus WCP 6.92 ± 0.61, CI 0.61-0.33, P < 0.05; and LSG 5.6 ± 0.47 versus WCP 6.5 ± 0.64, CI 1.04-0.73, P < 0.05, respectively (figure 1).

**Figure 2:**
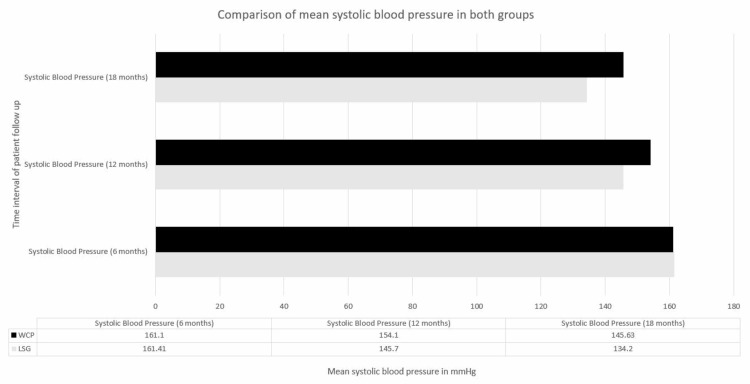
Comparison of mean systolic blood pressure in both groups WCP :Weight Control Program; LSG :Laparoscopic Sleeve Gastrectomy; mmHg: milimetre of mercury

There was no statistically significant difference between the groups for blood pressure at 6 months (LSG 161.41 ± 8.69 vs. WCP 161.1 ± 8.39, CI 2.0-2.5, P < 0.05), whereas at 12 and 18 months, there were statistically significant variations in blood pressure, with measurements of LSG 145.7 ± 7.6 versus WCP 154.1 ± 6.79, CI 10.3-6.1, P < 0.05 and LSG 134.2 ± 7.16 versus WCP 145.63 ± 5.94, CI 13.2-9.6, P < 0.05, respectively (figure 2).

**Figure 3:**
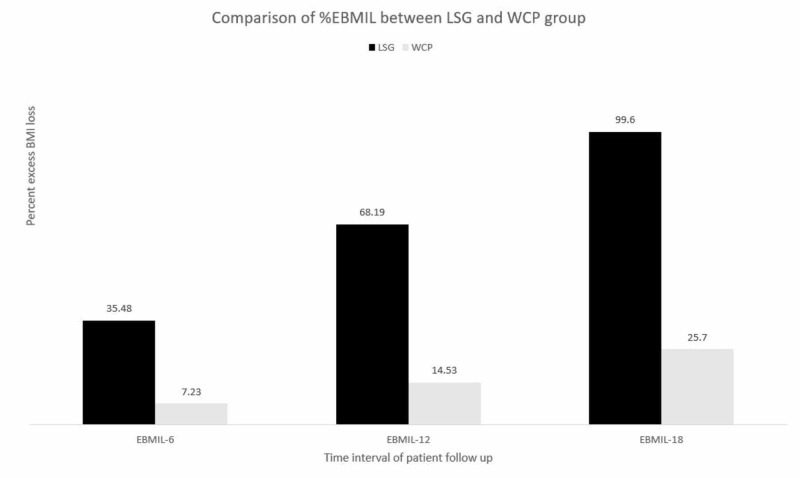
Comparison of %EBMIL between WCP and LSG group %EBMIL : Percent Excess Body Mass Index Loss; WCP: Weight Control Program; LSG: Laparoscopic Sleeve Gastrectomy

The Rosenberg SES levels increased in the entire study population. However, there was a considerably greater increase in Rosenberg scores at 18 months in the LSG group as compared to the WCP group (LSG 33.04 ± 3.44 vs. WCP 22.71 ± 1.81, CI 9.56-11.07, P < 0.05). Patient satisfaction scores were also better in the LSG group as compared to the WCP group (LSG 8.2 ± 0.84 vs. WCP 6.44 ± 0.91, CI 1.55-2.05, P < 0.05). %EBMIL at 6 months in the LSG group was 35.48%, compared to the WCP group at only 7.23%. At 12 months, %EBMIL had increased two-fold in the LSG group to 68.19%, while it was at 14.53% in the WCP group. At the final 18-month follow-up, %EBMIL in the LSG group was 99.60%, while it was only 25.70% in the WCP group (P < 0.05) (figure 3).

The statistical results of the study have been summarized in Table 1. 

**Table 1:** Mean measurements taken throughout the study period BMI: Body Mass Index; BP: Blood Pressure; CI: Confidence Interval; LSG: Laparoscopic Sleeve Gastrectomy; WCP: Weight Control Program

Mean Measurements	LSG Group	WCP Group	CI	P-Value
Age	41.7 years	38.8 years	41.72 – 39.41	0.01
Gender	Male: n = 34 Female: n = 72	Male: n = 33 Female: n = 70	1.6 – 1.7	0.01
Preoperative HBA1C	7.96%	7.87%	0.86 – 0.60	0.627
Preoperative Systolic BP	171.3 mmHg	169.8 mmHg	0.75 – 1.43	0.116
Initial BMI	33.3 kg/m^2^	32.5 kg/m^2^	0.49 – 1.10	0.05
BMI, 6 Months	30.4 kg/m^2^	32.0 kg/m^2^	1.85 – 1.29	0.05
BMI, 12 Months	27.6 kg/m^2^	31.4 kg/m^2^	4.12 – 3.47	0.05
BMI, 18 Months	25.0 kg/m^2^	30.6 kg/m^2^	5.94 – 5.24	0.05
HBA1C, 6 Months	7.26%	7.23%	0.13 – 0.18	0.72
HBA1C, 12 Months	6.45%	6.92%	0.61 – 0.33	0.05
HBA1C, 18 Months	5.68%	6.57%	1.04 – 0.73	0.05
Systolic BP, 6 Months	161.4 mmHg	161.1 mmHg	2.08 – 2.58	0.83
Systolic BP, 12 Months	145.7 mmHg	154.1 mmHg	10.30 – 6.44	0.05
Systolic BP, 18 Months	134.2 mmHg	145.6 mmHg	13.2 – 9.6	0.05
Self-Esteem Score, Day 0	14.9	14.7	0.69 – 0.99	0.73
Self-Esteem Score, 18 Months	33.0	22.7	9.56 – 11.00	0.05
Patient Satisfaction Score	8.26	6.44	1.57 – 2.05	0.05

## Discussion

The Pakistani population has witnessed an increase in the number of individuals suffering from obesity over time [6]. There has also been a rising trend in the use of bariatric surgery as a treatment modality for obesity in developing Asian countries since obesity has become a public health concern. The literature shows that the loss of excess weight has proven benefits in terms of the metabolic profile of patients suffering from obesity. Batsis et al. reported a significant reduction in the number of individuals with metabolic syndrome after bariatric surgery (from 87% down to 29%) [7]. There have also been studies proving the beneficial effects of weight loss for diabetes control. In our study population, the LSG group mean HBA1c level was reduced to a non-diabetic level after bariatric surgery. However, the mean HBA1c level in the WCP group was still in the diabetic range. In a meta-analysis of 357 patients, a significant decrease in HBA1c levels (2.59%) was noted among patients with Class I obesity who underwent bariatric surgery [8]. Another systematic review cited a linear relationship between the reduction of HBA1c levels and various weight loss methods, with an overall reduction of 0.1%/kg [9]. The results of these studies show an eminent superiority of bariatric surgery over WCPs with respect to glycemic control. Weight loss has also been reported to act on several neurohormonal mechanisms to control hypertension through, for instance, decreased oxidative stress, decreased insulin resistance, and decreased aldosterone release [10]. With regard to the comparison of hypertension as part of the metabolic profile, in our study, the majority of patients in the LSG group became normotensive by the 18-month follow-up (LSG 134.2 ± 7.16 mmHg), while in the WCP group (145.63 ± 5.94 mmHg), the majority still needed medication to control blood pressure. This is in agreement with a meta-analysis that included 14 studies (3,550 subjects) showing a resolution of hypertension in 62.17% and improvement in 35.70% of participants at the 5-year follow-up after sleeve gastrectomy [11]. Similarly, another meta-analysis of 25 studies found a direct link between weight loss and reduction of hypertension. Further, a decrease of 1 kg of weight has been associated with an approximate 1 mmHg decline in systolic blood pressure [12]. In the Asian population, individuals with Class I obesity who have undergone LSG have shown a greater %EBMIL than other treatment modalities [13]. In our study, the LSG group had a more rapid change in %EBMIL at 6,12, and 18 months and reached nearly 100%, in comparison to the WCP group, where the trend of %EBMIL was very gradual and only reached 25% by the end of 18 months. According to a meta-analysis of randomized controlled trials and cohort studies conducted in 2018, there was no high-quality evidence for treating obesity with WCPs because the level of benefits using such methods was minimal when contrasted with the weight loss that was needed [14]. However, one study noted that individuals using lifestyle modification to achieve weight loss surprisingly maintained 10% of excess weight loss throughout the duration of the study period [15]. This result did not relate to the main objective, but it shows that WCPs could help patients in controlling the disease. However, it must be taken into consideration that subjects treated with WCPs still have a BMI of 30 kg/m2 or more. A similar study comparing lifestyle intervention with bariatric surgery in Class I obesity patients showed similar results, with a %EBMIL of 16.20% and 17.90% at 1 and 3 years, respectively, in the lifestyle intervention group [15], which compares with a %EBMIL of 14.53% and 25.70% at 12 and 18 months, respectively, in our WCP group. Additionally, %EBMIL of 67.90% and 76.90% at 1 and 3 years, respectively, in their bariatric surgery group is comparable to our study’s %EBMIL of 68.19% and 99.60% at 12 and 18 months, respectively, in the LSG group. Obesity has a tremendous social and psychological impact as well. The Rosenberg SES scores were initially 15.53 ± 3.39 in our LSG group and 14.77 ± 2.64 in our WCP group. At the third follow-up (18 months), there had been a significant rise in self-esteem scores in the LSG group to 33.04 ± 3.44. Similarly, a prospective cohort study involving 32 individuals undergoing sleeve gastrectomy reported a significant rise in self-esteem scores (P value = 0.003) after 1 year [16]. A study of obese women by Dennis et al. also reported improved self-esteem and mood after a structured WCP, in agreement with our study [17]. Furthermore, the overall patient satisfaction in our LSG group was comparable to a large cohort study of 110 sleeve gastrectomy patients (8.2/10 vs. 8/10, respectively) [18].

The limitations of our study include smaller sample size and a short follow-up duration. However, because this is the first study from Pakistan on this topic, our results make a vital contribution to the literature. Additionally, we plan to do an extended follow-up study with a larger cohort of patients.

## Conclusions

Our study elucidates a clear superiority of LSG over a structured WCP with regard to weight reduction, improvement in glycemic control, and reduction in blood pressure among Class I obesity patients. Additionally, this study showed that patients reported markedly improved self-esteem and satisfaction after bariatric surgery as compared to lower levels of improvement with a WCP, pointing to bariatric surgery as a preferred mode of treatment for Class I obesity.

## References

[1] Cefalu WT, Bray GA, Home PD (2015). Advances in the science, treatment, and prevention of the disease of obesity: reflections from a Diabetes Care editors’ expert forum. Diabetes Care.

[2] Inge TH, Krebs NF, Garcia VF (2004). Bariatric surgery for severely overweight adolescents: concerns and recommendations. Pediatrics.

[3] Dietrich A, Aberle J, Wirth A, Müller-Stich B, Schütz T, Tigges H (2018). Obesity surgery and the treatment of metabolic diseases. Dtsch Arztebl Int.

[4] Tham KW, Lee PC, Lim CH (2019). Weight management in obstructive sleep apnea: medical and surgical options. Sleep Med Clin.

[5] Buchwald H (2014). The evolution of metabolic/bariatric surgery. Obes Surg.

[6] Shah AA, Shariff AH (2013). Obesity and the need for bariatric surgery in Pakistan. Asian J Endosc Surg.

[7] Batsis JA, Romero-Corral A, Collazo-Clavell ML, Sarr MG, Somers VK, Lopez-Jimenez F (2008). Effect of bariatric surgery on the metabolic syndrome: a population-based, long-term controlled study. Mayo Clin Proc.

[8] Li Q, Chen L, Yang Z (2012). Metabolic effects of bariatric surgery in type 2 diabetic patients with body mass index < 35 kg/m2. Diabetes Obes Metab.

[9] Gummesson A, Nyman E, Knutsson M, Karpefors M (2017). Effect of weight reduction on glycated haemoglobin in weight loss trials in patients with type 2 diabetes. Diabetes Obes Metab.

[10] Cohen JB (2017). Hypertension in obesity and the impact of weight loss. Current Cardiol Rep.

[11] Graham C, Switzer N, Reso A (2018). Sleeve gastrectomy and hypertension: a systematic review of long-term outcomes. Surg Endosc.

[12] Sabaka P, Dukat A, Gajdosik J, Bendzala M, Caprnda M, Simko F (2017). The effects of body weight loss and gain on arterial hypertension control: an observational prospective study. Eur J Med Res.

[13] Ismail M, Nagaraj D, Rajagopal M, Ansari H, Nair M, Hegde A, Rekha PD (2019). Seven-year outcomes of laparoscopic sleeve gastrectomy in Indian patients with different classes of obesity. Obes Surg.

[14] Solmi M, Köhler CA, Stubbs B (2018). Environmental risk factors and nonpharmacological and nonsurgical interventions for obesity: an umbrella review of meta‐analyses of cohort studies and randomized controlled trials. Eur J Clin Invest.

[15] Vitiello A, Angrisani L, Santonicola A, Iovino P, Pilone V, Forestieri P (2019). Bariatric surgery versus lifestyle intervention in class I obesity: 7-10-year results of a retrospective study. World J Surg.

[16] Aldaqal SM, Sehlo MG (2013). Self-esteem and quality of life in adolescents with extreme obesity in Saudi Arabia: the effect of weight loss after laparoscopic sleeve gastrectomy. Gen Hosp Psychiatry.

[17] Dennis KE, Goldberg AP (1996). Weight control self-efficacy types and transitions affect weight-loss outcomes in obese women. Addict Behav.

[18] Arman GA, Himpens J, Dhaenens J, Ballet T, Vilallonga R, Leman G (2016). Long-term (11+ years) outcomes in weight, patient satisfaction, comorbidities, and gastroesophageal reflux treatment after laparoscopic sleeve gastrectomy. Surg Obes Related Diseases.

